# Zinc Oxide Quantum Dots May Provide a Novel Potential Treatment for Antibiotic-Resistant *Streptococcus agalactiae* in *Lama glama*

**DOI:** 10.3390/molecules28135115

**Published:** 2023-06-29

**Authors:** Ziyao Zhou, Ting Zhang, Yixin Chen, Xiaoxiao Zhou, Yalin Zhong, Haifeng Liu, Zhijun Zhong, Yanchun Hu, Fei Liao, Xianxiang Wang, Guangneng Peng

**Affiliations:** 1 College of Veterinary Medicine, Sichuan Agricultural University, Chengdu 611130, China; zzhou@sicau.edu.cn (Z.Z.); 410140017@163.com (H.L.);; 2 Chengdu Center for Animal Disease Prevention and Control, Chengdu 610041, China; spongebob19891104@gmail.com; 3 Guizhou Vocational College of Agriculture, Qingzhen 551400, China; 4 College of Science, Sichuan Agricultural University, Chengdu 611130, China

**Keywords:** *Streptococcus agalactiae*, antibiotic resistance, zinc oxide quantum dots

## Abstract

*Streptococcus agalactiae* is a significant pathogen that can affect both human beings and animals. The extensive current use of antibiotics has resulted in antibiotic resistance. In our previous research, we found that zinc oxide quantum dots (ZnO QDs) had inhibitory effects on antibiotic-resistant microorganisms. In this study, a strain of *Streptococcus agalactiae* *WJYT1* with a broad antibiotic-resistant spectrum was isolated and identified from *Lama glama* at Sichuan Agricultural University Teaching Animal Hospital. The genome for the resistance and virulence genes was analyzed. Additionally, the antibacterial effects and anti-virulence mechanism of ZnO QDs for *S. agalactiae* *WJYT1* were investigated. The results showed that the genome of *S. agalactiae* *WJYT1* is 1,943,955 bp, containing 22 resistance genes and 95 virulence genes. ZnO QDs have a good antibacterial effect against *S. agalactiae* *WJYT1* by reducing bacterial growth and decreasing the expression of virulence genes, including *bibA*, *hylB*, *sip*, and *cip*, which provides a novel potential treatment for *S. agalactiae*.

## 1. Introduction

*Streptococcus agalactiae*, commonly known as group B *Streptococcus*, was first identified by Rebecca Lancefield in the 1930s [1]. This pathogen can affect both humans and animals and is associated with various conditions, including mastitis, caseous lymphadenitis, infectious skin necrosis, and purulent infections in *Lama glama* [2,3]. Recently, antibiotic resistance has become a growing concern [4,5]; microbiome adaptability, pathogenicity, and transmissibility [6], posing a threat to both the environment and human health. Therefore, there is an urgent to explore a novel antibacterial strategy.

In recent years, biomedical nanomaterials have attracted great attention because of their outstanding biological characteristics and wide application prospects. At present, novel nanocomposites, such as ZnO [7], CuO/ZnCdS [8], Mn_2_CuO_4_/CdO [9], and Ag/Cu_2_MoO_4_ [10] nano-materials, have made great breakthroughs in the inhibition of bacteria and the treatment of diseases. Among them, zinc oxide quantum dots (ZnO QDs) have interested researchers due to their excellent antibacterial effect [11,12]. Relevant studies have shown that the main antimicrobial toxicity mechanism of ZnO QDs is based on their ability to induce excess ROS production [13] and the cumulative release of Zn^2+^ [14], which can prevent the formation and spread of bacterial biofilms, leading to bacterial death [15,16]. Our previous research found that ZnO QDs had an inhibitory effect on antibiotic-resistant microorganisms [17,18].

Virulence genes and antibiotic resistance genes are crucial in understanding the pathogenic and antibiotic resistance mechanisms of pathogens. However, there is little research on ZnO QDs for *S. agalactiae* virulence and antibiotic resistance genes. In this study, a strain of *S. agalactiae* *WJYT1* with muti-antibiotic-resistance from a sick llama (*Lama glama)* at Sichuan Agricultural University Teaching Animal Hospital was isolated and identified. The aim of this study was to identify the virulence and antibiotic resistance genes of *S. agalactiae* *WJYT1* as well as the potential interventional effect of ZnO QDs in treating *S. agalactiae* infections.

## 2. Results

### 2.1. Results for the Isolation and Identification of S. agalactiae

**Figure 1:**
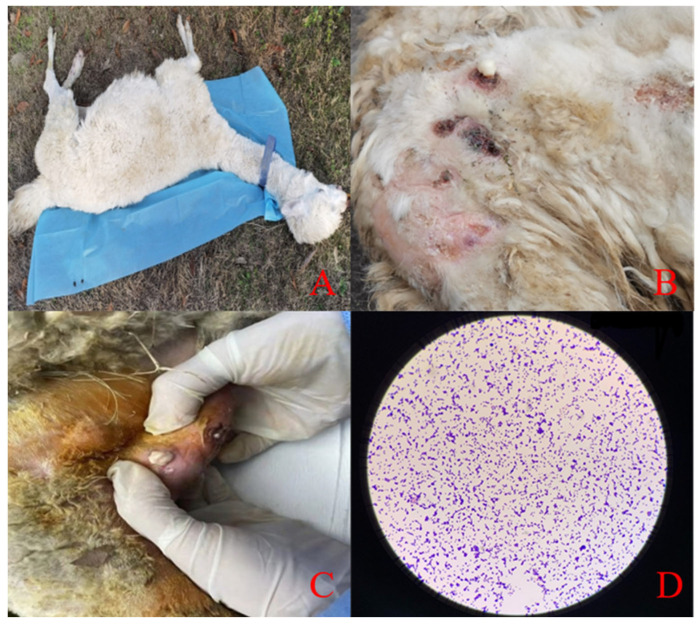
(**A**) The general photo of the clinical case of a llama. (**B**) Superficial abscesses. (**C**) White thick juice was observed by squeezing the abscess site. (**D**) Isolated strain, Gram stain, 40×.

The sick *Lama glama* showed an increased body temperature, confusion, depression, decreased appetite, and decreased activity. A physical examination of its eyes, ears, and nasal cavity showed no obvious secretions. The palpation of its superficial lymph nodes was enlarged. The auscultation of its chest cavity had no obvious murmur while its heart rate was accelerated. The *Lama glama* had a disheveled coat and septic infections on all its extremities. White cheese-like nodules were observed after squeezing the abscess. The results of the microscopic examination of the isolated strain showed single or chain Gram-positive bacteria microscopically (Figure 1).

### 2.2. Molecular Identification Results

According to the 16S rRNA alignment, the isolated strain was distinguished as *S. agalactiae*, specifically *S. agalactiae* *WJYT1*. Its sequence was uploaded to the NCBI database with the accession number OQ930763.

### 2.3. Identification of Antibiotic Susceptibility

Our antibiotic resistance tests showed that *S. agalactiae* *WJYT1* is resistant to all tested antibiotics, including aminoglycosides, β-lactams, sulfonamides, chloramphenicol, quinolones, glycylcyclines, tetracyclines, glycopeptides, and lincosamides (Table 1).

### 2.4. High-Throughput Sequencing Results

The original data were 1217 MB, and after data processing, they were filtered down to 1062 MB clean data. After genome assembly, the total genome length was 1,943,955 bp, with a GC ratio of 35.22%. According to their alignment, 6736 genes were mapped on the GO database. Specifically, 3328 genes were enriched in biological processes, many of which are related to cellular processes, metabolic processes, and localization (Figure S1). The KEGG metabolic classification showed that 63 genes were enriched in human disease, with many associated with antibiotic resistance, immune diseases, and infectious diseases (Figure S2). Similarly, through the analysis of the COG database, a total of 24 functional annotations were obtained (Figure S3). Among them, most of the gene clusters (containing 205 genes) were involved in translation, ribosomal structure, and biogenesis. The second largest category was carbohydrate transport and metabolism, with 167 genes involved.

### 2.5. Virulence and Pathogenic Analysis

Through their alignment in the VFDB database, a total of 95 virulence genes were identified, including the following: capsule-related genes (23 genes such as *neuA*, *neuC*, and *neuD*), beta-hemolysin-related genes (9 genes such as *cylX*, *cylD*, *cylG*), group B *Streptococcus* immunogenic bacterial adhesin-related genes (such as *bibA*), bacterial surface immunogenic protein-related genes (such as *sip*), hyaluronidase-related genes (such as *hylB*), and CAMP factor-related genes (such as *cfb*). The specific results are shown in Table 2.

### 2.6. Analysis of Antibiotic Resistance Genes of S. agalactiae

The results of the resistance gene information annotated in the CARD database are shown in Table 3, of which the main mechanisms of resistance are as follows: antibiotic target alteration, antibiotic efflux, antibiotic target replacement, antibiotic inactivation. The other database of the ARDB results is listed in Supplementary Materials Table S1.

### 2.7. Effect of ZnO QDs on S. agalactiae

**Figure 2:**
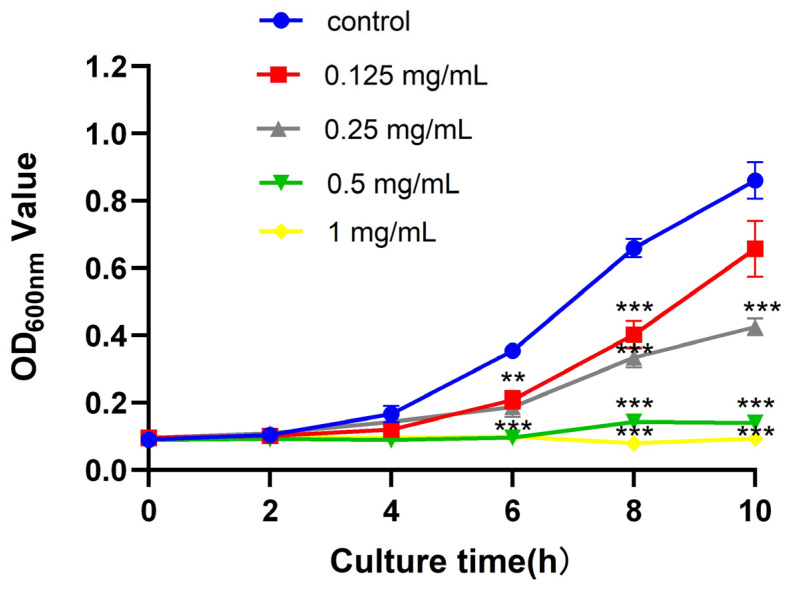
Growth curves of *S. agalactiae* lactis under the action of different concentrations of nano-zinc oxide. Error bars refer to the SD of 3 replicates of each assay (** *p* < 0.01, *** *p* < 0.001).

Through the plate coating experiment, the ZnO QDs were shown to have an inhibitory effect on the isolated antibiotic-resistant *S. agalactiae* *WJYT1* with a minimum inhibitory concentration of 0.5 mg/mL (Figure S4). The growth curve experiment revealed that the antimicrobial effect of the ZnO QDs was insignificant within the first 2 h (Figure 2). After 10 h, *S. agalactiae* *WJYT1* was significantly inhibited by the ZnO QDs with concentrations of 0.25–1 mg/mL decreasing by 49.17%, 78.3%, and 88.01% (*p* < 0.001), compared to those of the control group, respectively. There was no significant difference between the group with 0.125 mg/mL and the control group (*p* > 0.05).

### 2.8. Effect of ZnO QDs on Virulence Genes of S. agalactiae

**Figure 3:**
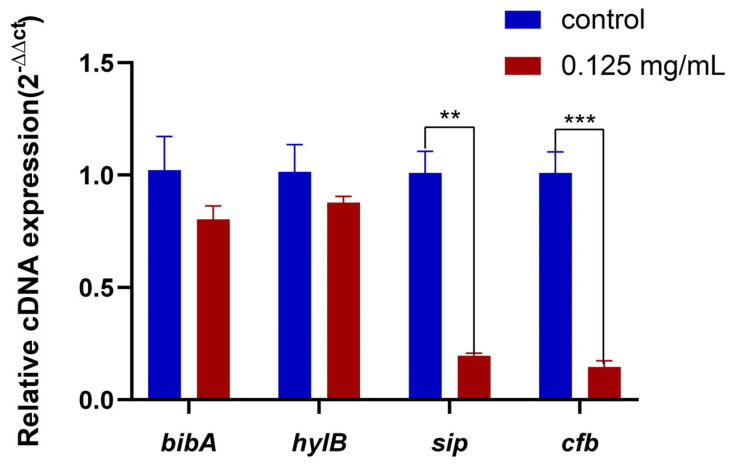
Changes in the expression levels of *bibA*, *hylB*, *sip*, and *cfb* genes after different concentrations of ZnO QDs acting on *S. agalactiae* (** *p* < 0.01, *** *p* < 0.001). Error bars refer to the SD of 3 replicates of each assay.

When the concentration of the ZnO QDs was 0.125 mg/mL (1/4 MIC), the expression levels of *sip* and *cfb* were significantly inhibited (Figure 3). Although there was no significant difference between the *hylB* and *bibA* genes, the transcription levels were reduced by 21.4% and 13.5%, respectively, compared to those of the control group.

## 3. Discussion

*S. agalactiae* is a pathogenic bacterium that affects both animals and humans and is a common cause of neonatal sepsis, mastitis, sepsis, and other diseases [19]. In this study, we isolated a highly antibiotic-resistant strain of *S. agalactiae* from a sick *Lama glama* in an animal hospital in Sichuan Province, China. The llama had a large abscess on the surface of its skin and had been treated with multiple ineffective antibiotics, indicating severe systemic sepsis due to *S. agalactiae* infection.

β-lactams, aminoglycosides, macrolides, lincosamides, fluoroquinolones, and tetracyclines are all first-line antibiotics for the treatment of *S. agalactiae* [20,21]. Penicillin antibiotics are the drug of choice for the treatment of *S. agalactiae* infections, which are typically highly sensitive to this class of antibiotics [22]. According to the treatments in the other animal hospital, the attending physicians had failed to treat the animal using the antibiotics enrofloxacin, amikacin, and meropenem. Our susceptibility results showed that the isolated *S. agalactiae* strain was resistant to aminoglycosides, β-lactams, sulfonamides chloramphenicol, quinolones, glycylcyclines, tetracyclines, glycopeptides, and lincosamides. Using NGS, the genes related to antibiotic resistance were found to be *pbp2x*, *rpoB*, and *mexD*, consistent with the resistance phenotypes. Meanwhile, related studies have shown the potential for the horizontal transfer of several of these genes [23,24,25]. Notably, we identified the *pbp2x* gene, a penicillin-binding protein involved in the final stages of peptidoglycan assembly and essential for bacterial growth and survival [26]. Our analysis of *S. agalactiae* with reduced penicillin sensitivity and insensitivity to fluoroquinolone drugs suggests possible amino acid mutations in penicillin-binding proteins such as *pbp2x* [27]. We also discovered the existence of *rpoB*, coding for the RNA polymerase β subunit, which is the target of rifampicin [28]. An essential drug in the treatment of tuberculosis and other mycobacterial infections, more than 95% of RIF-resistant mutations are associated with mutations in the *rpoB* gene [27]. The isolated strain’s resistance to rifamycin may be related to the expression regulation of *rpoB*. In addition, our analysis revealed the presence of *mexD*, a member of the resistance-nodulation-division (RND) antibiotic efflux pump MexCD-OprJ. Previous studies have shown that the MexCD-OprJ efflux pump is associated with multiantibiotic resistance [29]. The isolated strain’s resistance to multiple antibiotics may be related to the expression regulation of *mexD*. Further analyses are needed to confirm this conjecture.

*S. agalactiae* is a main pathogen in *Lama glama* mastitis, caseous lymphadenitis, infectious skin necrosis, and purulent infection [3]. To detect virulence genes, the genome of *S. agalactiae* *WJYT1* was aligned to the VFDB database. The resulting analysis revealed the presence of several virulence genes, including *hylB*, *cfb*, *sip*, and *bibA*. Among these genes, *hylB* is particularly important as it plays a crucial role in invading the host and evading host immunity [30]. *hylB*’s primary mechanism is related to the degradation of hyaluronic acid, which enables the colonization and invasion of pathogenic bacteria at the epithelial barrier, thereby contributing to the pathogenesis of infection [31]. *Cfp* is a CAMP factor, which is closely related to the virulence and pathogenicity of *S. agalactiae* [32]. It has been shown to form discrete transmembrane pores in cell membranes, causing the lysis of components of the cell membrane, damaging host antibodies and weakening host immunity [33,34]. The pathogenicity of *S. agalactiae* is mainly caused by virulence factors and surface proteins. Surface immunogenic protein (Sip) is an *S. agalactiae* surface-exposed protein found to be present in every serotype of *S. agalactiae* isolates and highly conserved [35].

ZnO is a material with excellent antibacterial activity in many drug-resistant pathogens, including *S. aureus*, *Escherichia coli* (*E. coli*) [36], and *Candida albicans* [37]. Compared to traditional metal bacteriostatic materials, when the size of ZnO is reduced to the nanometer range, ZnO QDs typically exhibit additional morphologies and significant antibacterial activity against various bacterial species, e.g., *E. coli*, *S. aureus*, and *Salmonella pullorum*, which have been explored by numerous researchers [18,38,39,40], prompting us to explore whether they also have inhibitory effects on drug-resistant *S. agalactiae* in our study. Our experimental results showed that the inhibition ability of ZnO QDs for *S. agalactiae* *WJYT1* was concentration dependent. In our study, the MIC of ZnO QDs for *S. agalactiae* *WJYT1* was lower than that for *E. coli* while higher than that for *S. aureus* [18]. This gap may due to the strong resistance for the isolated bacterial species in our study. Although ZnO QDs at 1/4 MIC could not significantly inhibit the growth of *S. agalactiae*, the experimental results showed that it could significantly inhibit the expression of partial *S. agalactiae* virulence genes, such as *sip* and *cfb*. The pathogenic mechanism of *S. agalactiae* relies mainly on its ability to adhere and invade host cells, evade phagocytosis and immune clearance, and eventually produce pathogenicity [41]. The pathogenicity of *S. agalactiae* depends mainly on its virulence genes, of which *cfp* and *sip* can help the bacterium invade and evade host immunity [41]. Our experimental results showed that ZnO QDs can inhibit partial virulence without inhibiting growth. Therefore, it is speculated that ZnO QDs may affect the ability of *S. agalactiae* to invade host cells and evade host immunity by inhibiting the expression of these virulence genes. The effect mechanism of ZnO QDs on bacteria may include the following: (1) the smaller particle size of ZnO QDs makes it easier to attach to the bacterial surface, which is conducive to reactive oxygen species (ROS) and Zn^2+^ entering the bacterial body, thereby producing beneficial antibacterial activity; and (2) ZnO QDs can interact with the surface of bacteria, even entering the cell nucleus, interfering with bacterial biochemical reactions [42]. Interestingly, some research reports have shown that ZnO QDs are non-toxic to animal cells with good biocompatibility, while harmful to microorganisms, indicating their potential use as antibacterial agents [43]. In our study, the decrease in the invading and evading genes proved the effect of the ZnO QDs on *S. agalactiae*. However, the specific mechanism of action in *Lama glama* requires further in-depth research.

Llamas have a high economic value due to their fur, meat, skin, and milk. In recent years, llamas have become highly social animals and live in animal parks, attracting large numbers of tourists. However, close contact with llamas increases the risk of transmission of zoonotic diseases. Although currently no human infection with *S. agalactiae* has been reported, the potential for the transmission of *S. agalactiae* from animals to humans has been briefly described in cattle [44]. Therefore, there may be a possible link between *Lama glama* exposure and *S. agalactiae* in humans. Horizontal transfer is an important mechanism for strengthening antibiotic resistance and enriching resistance genes. We isolated a highly antibiotic-resistant strain in *Lama glama*, which indicated that it may have shared horizontal metastasis with many resistant bacteria. The llama in our study came from a local alpaca park, but it was kept alone and not in contact with other animals, so the possibility of human movement causing the transfer of resistant genes cannot be ruled out. Therefore, we should pay more attention to the prevention and control of group B *Streptococcus* in the future.

## 4. Materials and Methods

### 4.1. Basic Information of Animals

In November 2021, a sick llama (*Lama glama)* was brought to Sichuan Agricultural University for treatment. The llama had been treated with various antibiotics, including enrofloxacin, amikacin, and meropenem previously in another animal hospital. The sick llama showed symptoms of sepsis, including increased body temperature, confusion, depression, decreased appetite, and decreased activity.

### 4.2. Isolation and Identification of S. agalactiae

Samples were collected from lesions in the extremities and streaked in Luria-Bertani (LB) medium with 5% defibrous sheep blood, incubated at 37 °C for 48 h. Individual colonies with different morphologies were selected and purified through three continuous passages on blood agar. Purified isolates were stored at −80 °C in 50% glycerol for subsequent experiments. Gram staining was performed to identify bacterial morphology.

### 4.3. Molecular Identification

DNA of the bacterial isolates was extracted using a DNA Extraction Kit (Tiangen, Beijing, China) and measured by ND-1000 microUV spectrophotometer (NanoDrop Technologies, Wilmington, DE, USA). The primers 27F (5′-AGAGTTTGATCCTGGCTCAG-3′) and 1492R (5′-GGTTACCTTGTTACGACTT-3′) were used to amplify 16S rDNA gene. PCR reactions (25 μL) contained 12.5 μL PCR Master Mix, 9.5 μL nuclease-free H_2_O, 1 μL forward primer, 1 μL reverse primer, and 1 μL DNA sample. The PCR procedure was performed as follows: predenaturation at 94 °C for 5 min, followed by 30 cycles (30 s of denaturation at 94 °C, 30 s of annealing at 55 °C, and 1 min of extension at 72 °C), with a final extension at 72 °C for 7 min. The PCR products were stored at 4 °C for subsequent checking on 2% agarose gel and sent to Sangon Biotech Co. Ltd. (Shanghai, China) for sequencing. A homology search was performed by Blast function in the GenBank database (http://www.ncbi.nlm.nih.gov/BLAST, accessed on 22 November 2021).

### 4.4. Antibiotic Susceptibility Testing

The disc-diffusion test was used to assess the antibiotic susceptibility of the bacteria isolated above. Fourteen antimicrobials were tested, including amikacin, gentamicin, meropenem, cefepime, ampicillin, amoxicillin, ceftriaxone, cefoperazone, chloramphenicol, ciprofloxacin, tigecycline, doxycycline, vancomycin, and clindamycin. The diameters (mm) of the inhibition zones were measured to classify the antibiotic susceptibility as resistance (R), moderate susceptibility (MS), or susceptibility (S) based on the parameters of the Clinical and Laboratory Standards Institute [45].

### 4.5. High-Throughput Sequencing

The purified DNA was sent to Novogene Co. Ltd. (Beijing, China) for Hi-seq 2000 Next Generation Sequencing (NGS). After sequencing, clean data were obtained, assembled by SOAP denovo (version 2.04) [46,47], SPAdes (version 3.6.2) [48], and AbySS (version 2.0) [49], and finally integrated by CISA (version 1.3) [50]. The protein sequences of the predicted genes were compared to the GO, KEGG, and COG databases as well as the classic protein function database using Diamond (version 2.1.8) [51]. The amino acid sequences were aligned to VFDB (version 2022) and ARDB (version 1.1) [52] databases using Diamond. The amino acid sequences of target species were compared with the CARD database (version 3.2.2) using Resistance Gene Identifier (RGI) software provided by the CARD database, which obtained annotated results [53]. The raw data were uploaded to NCBI database with the accession number PRJNA970803.

### 4.6. Antibacterial Effect of ZnO QDs

To investigate the effect of ZnO QDs on *S. agalactiae*, we conducted a series of experiments. The enriched bacterial solution was firstly adjusted to a concentration of 1 × 10^8^ CFU/mL. The microbroth dilution method was then performed to determine the minimum inhibitory concentration (MIC). The prepared ZnO QDs were added to the bacterial solution to obtain concentrations of 0, 1/4, 1/2, 1, and 2 MIC, respectively. The growth curve was measured for 10 h in each group cultured in a shaker at 37 °C.

### 4.7. Detection of Virulence Genes

To further investigate the effect of ZnO QDs on virulence genes, four virulence genes including *bibA*, *hylB*, *sip*, and *cip*, representing adhesion and colonization, invasion, and immune evasion, were selected. ZnO QDs with a concentration of 1/4 MIC and no significant inhibitory effect at 10 h were added to the bacterial solution and incubated at 37 °C for 24 h. Bacterial total RNA was extracted using an RNA extraction kit (Tiangen, Beijing, China). Reverse transcription was performed with a reverse transcription kit (Tsingke, Beijing, China). The mRNA expression of virulence genes was detected by qPCR using SYBR Green dye (Servicebio, Wuhan, China). IBM SPSS Statistics (version 27) was used for statistical analysis.

## 5. Conclusions

*S. agalactiae* is a kind of conditional pathogen, which can cause serious infection when the body’s immunity is decreased. Currently, antibiotic resistance is increasing the urgency of finding alternative treatment methods. Our study illustrated that a biomedical nanomaterial, ZnO QDs, could inhibit the growth of *S. agalactiae* with a broad antibiotic-resistant spectrum isolated from *Lama glama*, further reducing the expression of the virulence gene even with a low concentration. Therefore, ZnO QDs are expected to become a potential alternative for antibiotic-resistant bacteria.

## Figures and Tables

**Table 1:** Antimicrobial resistance profile of *S. agalactiae*.

Antibiotic Class	Antimicrobial	SIR
Aminoglycosides	Amikacin	R
	Gentamicin	R
β-lactams	Meropenem	R
	Cefepime	R
	Ampicillin/Sulbactam	R
	Amoxicillin/Clavulanate	R
	Ceftriaxone	R
	Cefoperazone	R
Chloramphenicols	Chloramphenicol	R
Quinolones	Ciprofloxacin	R
Glycylcyclines	Tigecycline	R
Tetracyclines	Doxycycline	R
Glycopeptides	Vancomycin	R
Lincomycins	Clindamycin	R

SIR sensitive (S), intermediate sensitive (IS), resistant (R).

**Table 2:** Classification of virulence genes in VFDB database.

VF Name	Related Genes
Capsule	*neuA*, *neuD*, *neuC*, *neuB*, *cpsL*, *cpsK*, *cpsJ*, *cpsI*, *cpsH*, *cpsG*, *cpsF*, *cpsE*, *cpsD*, *cpsC*, *cpsB*, *cpsY*, *uppS*, *cpsB*, *rgpB*, *rmlA*, *rmlC*, *rgpG*, *oppF*
Beta-hemolysin	*cylX*, *cylD*, *cylG*, *acpC*, *cylZ*, *cylA*, *cylI*, *cylJ*, *cylK*
ABC transporter	*fbpC*, *fagC*, *aatC*
Periplasmic binding protein-dependent ABC transport systems	*vctC*, *vctG*, *vctD*
Type VII secretion system	*essC*, *esxA*
LPS	*acpXL*, *fabZ*
ClpE	*clpE*
Polysaccharide capsule	*galE*
Streptococcal enolase	*eno*
Mitogenic factor 2	*mf2*
PrrA/B	*prrA*
PEB1/CBF1	*pebA*
RegX3	*regX3*
Lipoprotein diacylyceryl transferase	*lgt*
SodB	*sodB*
Cytolysin	*cylR2*
PdgA	*pdgA*
PDH-B	*pdhB*
Lipoate protein ligase A1	*lplA1*
Exopolysaccharide	*mrsA*
Nucleoside diphosphate kinase	*ndk*
Sortase A	*srtA*
PhoP	*phoP*
Pyrimidine biosynthesis	*carB*
Lipoate protein ligase A1	*lplA1*
RTX toxin	*rtxB*
CAMP factor	*cfb*
Cytolysin	*cylR2*
(p)ppGpp synthesis and hydrolysis	*relA*
Laminin-binding protein	*lmb*
Neuraminidase	*nanA*
Trehalose-recycling ABC transporter	*sugC*
ClpC	*clpC*
D-alanine-polyphosphoribitol ligase	*dltA*
Streptococcal plasmin receptor/GAPDH	*gapA*
Glutamine synthesis	*glnA1*
C3-degrading protease	*cppA*
Polysaccharide capsule	*manA*
LisR/LisK	*lisR*
LOS	*orfM*
Fibronectin-binding protein	*scpB*
ClpP	*clpP*
Pneumococcal surface antigen A	*psaA*
Streptococcal lipoprotein rotamase A	*slrA*
SigA	*sigA*
PI-2b pili	*lep*
Lipoprotein-specific signal peptidase II	*lspA*
Hemolysin III	*hlyIII*
Hyaluronidase	*hylB*
Fibronectin-binding proteins	*pavA*
Serine protease	*htrA*
Phytotoxin phaseolotoxin	*argK*
MprA/B	*mprA*
GroEL	*groEL*
Cytolysin	*cylR2*
Serine-threonine phosphatase	*stp*
Protein kinase G	*pknG*
Trigger factor	*ropA*
Listeria adhesion protein	*lap*
Surface immunogenic protein	*sip*
GbpC	*gbpB*
Polar flagella	*flmH*
Copper exporter	*ctpV*
Mitogenic factor 3	*mf3*
Cytolysin	*cylR2*
Group B *Streptococcus* immunogenic bacterial adhesin	*bibA*

**Table 3:** *S. agalactiae* antibiotic resistance genes and corresponding antibiotic resistance mechanisms.

Resistance Mechanism	Antibiotic Class	Antibiotic Resistance Ontology
antibiotic efflux	macrolide	*mtrA*
	nitroimidazole	*msbA*
	diaminopyrimidine	*oqxB*
	fluoroquinolone	
	glycylcycline	
	nitrofuran	
	tetracycline	
	fluoroquinolone	*efrB*
	macrolide	
	rifamycin	
	fluoroquinolone	*efrA*
	macrolide	
	rifamycin	
	fluoroquinolone	*adeH*
	tetracycline	
	aminoglycoside	*Pseudomonas*
	aminoglycoside	*MexD*
	aaminocoumarin	
	cephalosporin	
	diaminopyrimidine	
	fluoroquinolone	
	macrolide	
	phenicol	
	tetracycline	
	acridine dye	*arlR*
	isinfecting agents and intercalating dyes fluoroquinolone	
	fluoroquinolone	*norB*
		*pmrA*
	lincosamide	*lmrP*
	macrolide	
	streptogramin	
	tetracycline	
	acridine dye	*mdtN*
	disinfecting agents and intercalating dyes nucleoside	
antibiotic target alteration	lincosamide	*RlmA(II)*
	macrolide	
	peptide	*mprF*
	glycopeptide	*vanRF*
		*vanRM*
antibiotic inactivation	aminoglycoside	*AA* *′* *(6′)-Ip*
		*AAC(3)-Iib* *AN* *′* *(4* *′* *)-Ib*
antibiotic target alteration;antibiotic target replacement	peptide	*rpoB*
	rifamycin	
antibiotic target replacement	diaminopyrimidine	*DfrA42*

## Data Availability

The 16S rRNA sequencing data are available in the NCBI database with the accession number OQ930763. The genome raw data are available in the NCBI database with the accession number PRJNA970803.

## Supplementary Materials

The following supporting information can be downloaded at: https://www.mdpi.com/article/10.3390/molecules28135115/s1, Figure S1: *S. agalactiae WJYT1* gene function annotation GO functional classification chart; Figure S2: Functional annotation of the *WJYT1* gene of *S. agalactiae* KEGG metabolic pathway; Figure S3: *S. agalactiae WJYT1* gene function annotation COG function classification chart; Figure S4: ZnO QDs has an inhibitory effect on antibiotic-resistance *S. agalactiae* *WJYT1*; Table S1: *S. agalactiae* ARDB database alignment.
