# Supplementary material for: Zinc Oxide Quantum Dots May Provide a Novel Potential Treatment for Antibiotic-Resistant Streptococcus agalactiae in Lama glama
Source: Molecules. 2023 Jun 29;28(13):5115. doi: 10.3390/molecules28135115 (PMC10343708; doi:10.3390/molecules28135115)
Supplement: Supplementary file 1 [file molecules-28-05115-s001.zip › molecules-2447532-supplementary.pdf]

Supplementary

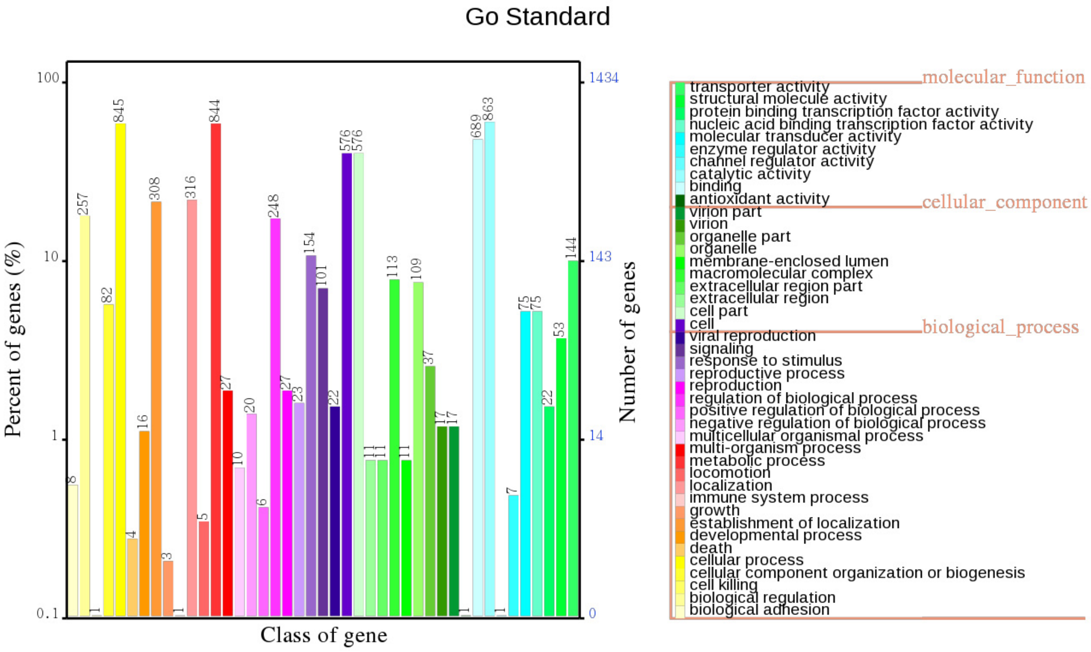

Figure S1 *S. agalactiae* WJYT1 gene function annotation GO functional classification chart.

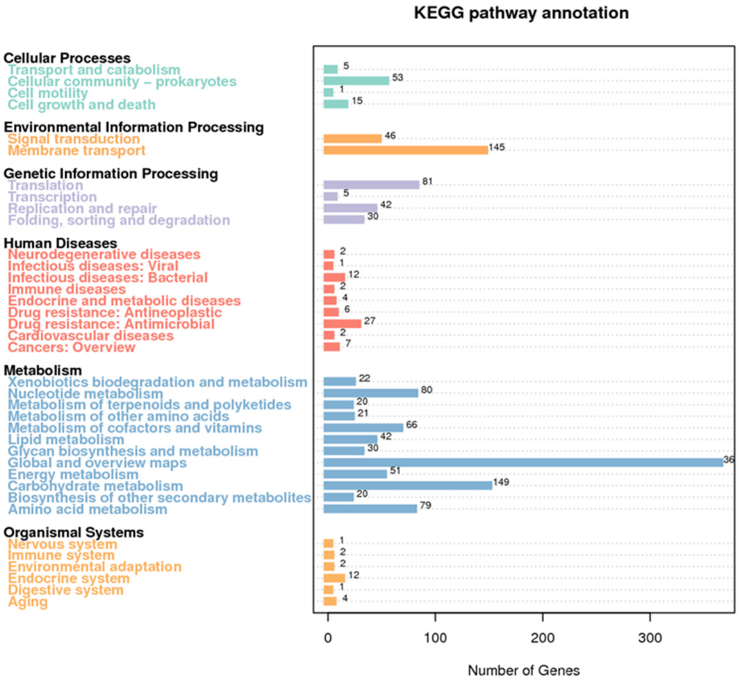

Figure S2 Functional annotation of the WJYT1 gene of *S. agalactiae* KEGG metabolic pathway.

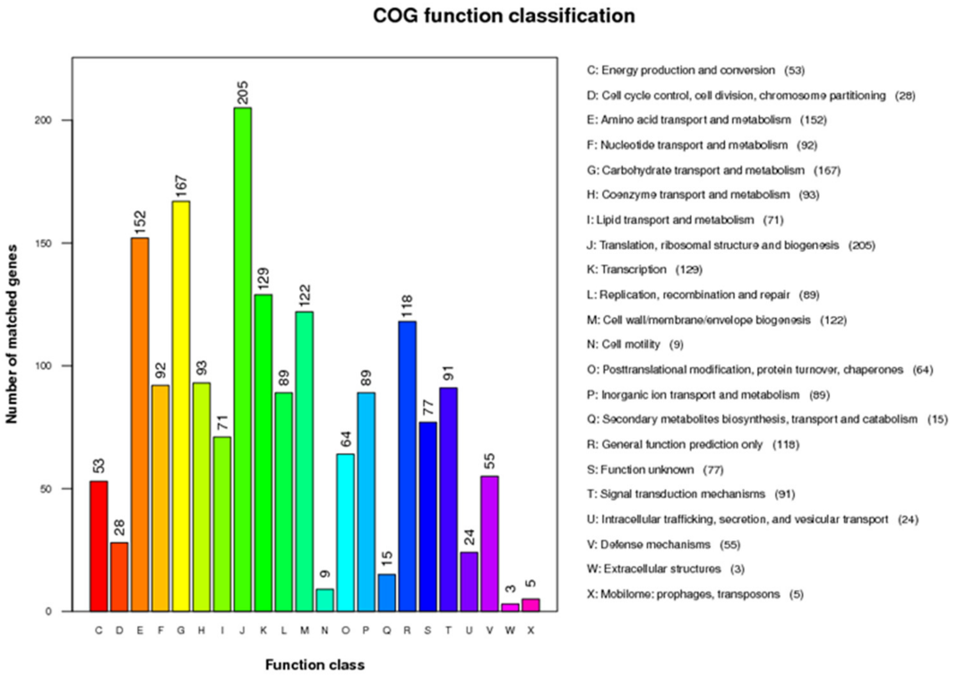

Figure S3 *S. agalactiae* WJYT1 gene function annotation COG function classification chart.

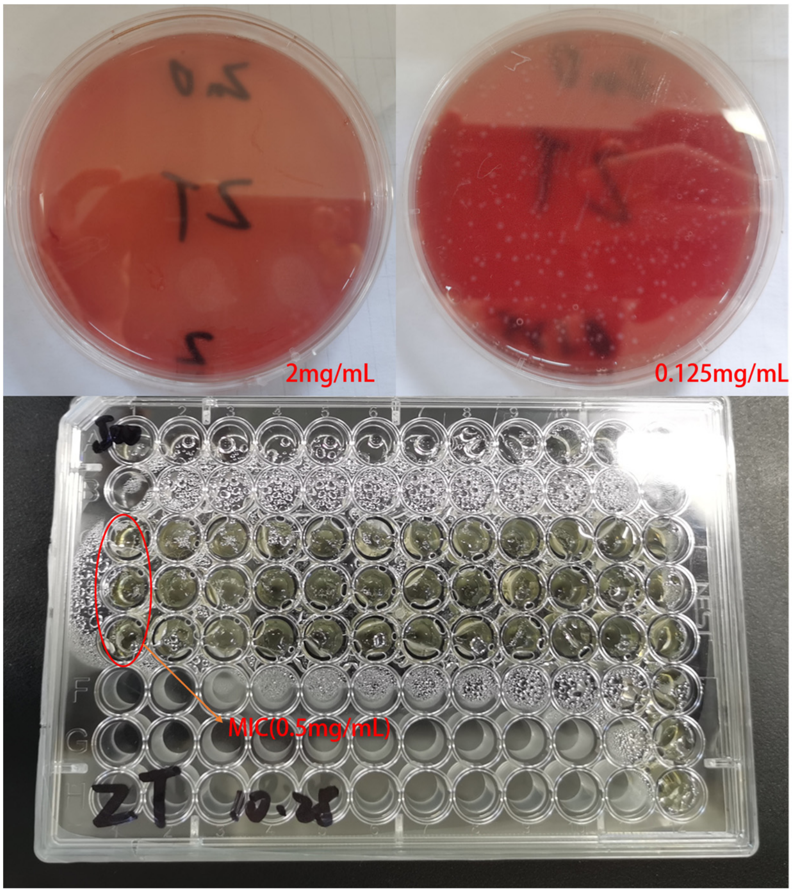

Figure S4 ZnO QDs has an inhibitory effect on antibiotic-resistance *S. agalactiae* WJYT1

Table S1 *S. agalactiae* ARDB database alignment

Resistance Type	Antibiotic Resistance	Description
vanre	vancomycin	VanE type vancomycin resistance operon genes, which can synthesize peptidoglycan with modified C-terminal D-Ala-D-Ala to D-alanine--D-serine.
vanrg	vancomycin	VanG type vancomycin resistance operon genes, which can synthesize peptidoglycan with modified C-terminal D-Ala-D-Ala to D-alanine--D-serine.
vanra	vancomycin,teicoplanin	VanA type vancomycin resistance operon genes, which can synthesize peptidoglycan with modified C-terminal D-Ala-D-Ala to D-alanine--D-lactate.
bcra	bacitracin	ABC transporter system, bacitracin efflux pump.
pmra	ciprofloxacin,norfloxacin	Major facilitator superfamily transporter. Multiantibiotic resistance efflux pump.
pbp2x	penicillin	The enzyme has a penicillin-insensitive transglycosylase N-terminal domain (formation of linear glycan strands) and a penicillin-sensitive transpeptidase C-terminal domain (cross-linking of the peptide subunits)
baca	bacitracin	Undecaprenyl pyrophosphate phosphatase, which consists in the sequestration of Undecaprenyl pyrophosphate.
